# Replacement of Wheat With Barley With or Without Exogenous Carbohydrases in Pacific White Shrimp (*Litopenaeus vannamei*): Effects on Growth, Digestibility, and Gene Expression

**DOI:** 10.1155/anu/4376710

**Published:** 2026-10-25

**Authors:** Jairo A. Gonzalez, Krishna P. Singha, Timothy J. Bruce, D. Allen Davis

**Affiliations:** ^1^ School of Fisheries, Aquaculture and Aquatic Sciences Auburn University Auburn 36849 Alabama USA auburn.edu

**Keywords:** β-glucanase, exogenous enzymes, nutrient digestibility, wheat replacement, xylanase

## Abstract

Barley can potentially replace wheat in aquaculture feed. However, barley contains relatively higher fiber and β‐glucans levels, which may reduce digesta viscosity, and hinder endogenous enzyme activities. Supplementation of exogenous enzymes in barley‐based diets could aid in hydrolyzing the non‐starch polysaccharides (NSP). To investigate the effects of enzyme supplements in barley‐based diets replacing wheat, a 5‐week growth trial was conducted on Pacific white shrimp (*Litopenaeus vannamei*). Six isonitrogenous (~35%) and isolipidic (~8%) diets were formulated, namely, whole wheat‐based (17.42%) basal diet (BW); barley‐based basal diet (BB); and four enzyme‐supplemented diets were BB supplemented with either xylanase (XYL) or a multienzymatic solution (MES) which contains pectinase, protease, phytase, β‐glucanase, XYL, cellulase, and amylase. XYL and MES amount was determined based on manufacturers’ recommended dose (low, L) (0.01 and 0.05 g/100 g diet, respectively) and double the recommended dose (high, H) (0.02 and 0.10 g/100 g diet, respectively) and denoted as BB:XYL‐L, BB:XYL‐H, BB:MES‐L, and BB:MES‐H. Shrimp (0.72 ± 0.03 g) were stocked in 108‐L aquaria. Diets were randomly assigned, with six tanks per treatment. At the completion of the trial, growth performance, apparent digestibility coefficients (ADCs), and physiological gene expression in hepatopancreas were analyzed. Growth parameters showed no significant differences (*p*  > 0.05). However, significant differences in ADC of energy (*p* = 0.010) and dry matter (*p* = 0.001) were observed. The BB:XYL‐L diet significantly increased energy digestibility compared to BB group. No significant differences in hepatopancreatic gene (*tor*, *tghsr1l*, *cat*, *tgfβ*, *propo*, *lgbp*, and *hsp70*) expression were detected among treatments (*p*  > 0.05), although clustering patterns suggested similarity between multienzyme and wheat‐based diets. Overall, barley can replace wheat in Pacific white shrimp diets without impairing growth, while XYL supplementation may improve energy utilization.

## 1. Introduction

Aquaculture represents one of the fastest‐growing sectors of global food production, with Pacific white shrimp (*Litopenaeus vannamei*) being among the most important crustacean species cultured worldwide [1]. Since formulated feed accounts for approximately 60% of production costs on shrimp farms, increased attention is being directed towards reducing feed costs to achieve economic sustainability. In shrimp nutrition, research has primarily focused on protein and lipid requirements and the selection of suitable sources for these nutrients, such as fishmeal and fish oil, which are predominantly derived from marine resources [2]. However, carbohydrates serve as the most inexpensive energy‐supplying nutrient, which spare protein use for growth via the protein‐sparing effect, thus reducing formulation costs [3, 4]. The ability of shrimp to utilize carbohydrates depends on the carbohydrate source, dietary inclusion level, processing procedures, degree of gelatinization, structural complexity, and the bioavailability of those carbohydrates [5–7]. Carbohydrates also play a functional role in feed processing by enhancing pellet‐forming ability and water stability, which are essential for maintaining feed quality in aquatic environments [8]. In Pacific white shrimp (*L. vannamei*), various cereal grains such as whole wheat flour, rice, and sorghum are commonly used in formulations and have shown positive outcomes in terms of energy digestibility and growth performance [4, 9]. However, the choice of carbohydrate‐rich ingredients in commercial shrimp feeds is frequently guided by price and supply considerations [10].

Different studies have shown that wheat meal often supports better weight gain and nutrient digestibility compared to other cereal meals in shrimp diets [2, 10]. However, given the importance of wheat in human foods, reliance solely on wheat may carry economic and supply risks driven by geopolitical events and price volatility [6]. Therefore, reducing dependence on wheat and evaluating alternative ingredients with similar nutritional composition, such as barley, are critical to mitigate these issues. Barley is a cereal grain with high market potential in poultry, swine, and fish diets [11]. High concentrations of β‐glucans in barley may enhance innate immune responses through their immunomodulatory properties. Pinedo‐Gil et al. [12] reported that including barley at up to 8% in rainbow trout (*Oncorhynchus mykiss*) diets can help reduce plasma cortisol levels. However, barley utilization in fish and shrimp diets remains constrained because of its higher crude fiber content (5%) compared to wheat (2.6%), low metabolizable energy, and the presence of antinutritional factors (ANFs) such as non‐starch polysaccharides (NSP) [13, 14]. Despite their immune properties, β‐glucans have been reported to increase the stickiness of the intestinal digesta, thereby decreasing nutrient absorption and negatively impacting growth in broilers [15] and pigs [16]. Therefore, dietary exogenous carbohydrases, such as β‐glucanase and xylan‐degrading enzymes, are commonly included in plant‐based feed to enhance digestibility. The enzymes disrupt NSP, releasing entrapped energy and increasing available energy for maintenance and growth [17, 18]. In pigs, a mix of β‐glucanase and β‐xylanase (XYL) increased the nutrient digestibility of barley‐based diets [19]. However, according to Stites et al. [20], there were no significant differences in growth in Florida pompano (*Trachinotus carolinus*) fed carbohydrase‐supplemented diets.

Several studies have evaluated the effect of different carbohydrate sources and carbohydrase supplementation on plant‐based diets fed to Pacific white shrimp on growth performance and nutrient utilization [2, 17]. However, results are inconsistent and data are limited. Accordingly, the present work evaluated the effects of barley as a wheat replacement and of carbohydrase supplements, including XYL and a multienzyme solution (containing β‐glucanase), on growth performance, nutrient utilization, and physiological gene expression in Pacific white shrimp. Although β‐glucan is the predominant NSP in barley, arabinoxylans are also important components of the barley cell wall that can limit nutrient accessibility. XYL was, therefore, selected to hydrolyze arabinoxylans and disrupt the cell‐wall matrix, whereas β‐glucanase activity was provided through the multienzyme solution. The enzyme supplementation strategy therefore compared the targeted activity of XYL against arabinoxylans with the broader activity of a multienzyme solution containing β‐glucanase and other carbohydrases. We hypothesized that barley unsupplemented diet would impair growth performance, nutrient utilization, and cause negative physiological effects, and carbohydrases supplementation would counter barley negative effects.

## 2. Materials and Methods

### 2.1. Feed Preparation

Six isonitrogenous (crude protein, CP, 35 g/100 g) and isolipidic (8 g/100 g) diets were prepared in the Aquatic Animal Nutrition Laboratory at the School of Fisheries, Aquaculture & Aquatic Sciences, Auburn University (Auburn, AL, USA), following standard procedures (Table 1). In the reference diet (BW), whole wheat (17.42 g/100 g diet) was used as the main carbohydrate source. In the barley‐based diet (BB), whole barley was used as a full replacement of whole wheat. The barley was used as is from the commercial source without any pre‐treatment. Four exogenous carbohydrases containing BB diets were formulated by supplementing either XYL at the manufacturer’s recommended low inclusion, 0.01% (BB:XYL‐L), and doubled as high level, 0.02% (BB:XYL‐H), or a multienzymatic solution (MES) at low level, 0.05% (BB:MES‐L), and high level, 0.10% (BB:MES‐H). XYL activity as declared by the manufacturer was 16,000 BXU/kg (Econase XT, AB vista., Marlborough, Wiltshire, UK), while the MES (Allzyme SFF, Alltech Inc., Nicholasville, KY, USA) contains pectinase (4,000,000 IU/kg), protease (700,000 IU/kg), phytase (300,000 IU/kg), β‐glucanase (200,000 IU/kg), XYL (100,000 IU/kg), cellulase (40,000 IU/kg) and amylase (30,000 IU/kg) as reported by Furuya et al. [21].

**Table 1:** Formulation (as is basis) of the experimental diets^a^ containing barley as a replacement of wheat, with or without enzyme supplementation and fed to Pacific white shrimp (*L. vannamei*) for 5‐weeks.

Ingredient (as is basis)	BW	BB	BB:XYL‐L	BB:XYL‐H	BB:MES‐L	BB:MES‐H
Whole wheat^b^	17.42	—	—	—	—	—
Whole barley^c^	—	17.42	17.42	17.42	17.42	17.42
Menhaden fishmeal^d^	6.00	6.00	6.00	6.00	6.00	6.00
Soybean meal^e^	51.10	52.00	52.00	52.00	52.00	52.00
Corn protein concentrate^f^	7.50	7.50	7.50	7.50	7.50	7.50
Menhaden fish oil^d^	2.47	2.47	2.47	2.47	2.47	2.47
Soy oil	3.70	3.63	3.64	3.63	3.63	3.63
Lecithin^g^	1.00	1.00	1.00	1.00	1.00	1.00
Cholesterol^h^	0.12	0.12	0.12	0.12	0.12	0.12
Corn starch^i^	5.59	4.76	4.74	4.74	4.71	4.66
Mineral premix^j^	0.50	0.50	0.50	0.50	0.50	0.50
Vitamin premix^k^	1.80	1.80	1.80	1.80	1.80	1.80
Choline chloride^l^	0.20	0.20	0.20	0.20	0.20	0.20
Stay‐C^m^	0.10	0.10	0.10	0.10	0.10	0.10
CaP‐dibasic^n^	2.50	2.50	2.50	2.50	2.50	2.50
Econase XT^o^	—	—	0.01	0.02	—	—
Allzyme SFF^p^	—	—	—	—	0.05	0.10
Proximate composition analysis (%)^q^						
Moisture	7.65	7.75	7.72	8.74	9.1	8.2
Crude protein	37.8	37.5	38.4	37.7	37.3	37.8
Lipids	9.26	8.88	8.78	9.07	8.8	8.82
Gross energy (kcal/kg)	4896.96	5012.81	5004.39	4504.84	4803.21	4871.46
Total ash	7.29	7.56	7.12	7.18	7.32	7.27

^a^Treatments: BW, wheat‐based diet; BB, barley‐based diet; BB:XYL‐L, barley‐based diet supplemented with 0.01% Econase XT25; BB:XYL‐H, barley‐based diet supplemented with 0.02% Econase XT25; BB:MES‐L, barley‐based diet supplemented with 0.05% allzyme SFF; and BB:MES‐H, barley‐based diet supplemented with 0.1% allzyme SFF.

^b^Bob’s Red Mill, Milwaukie, OR, USA.

^c^Rangen group, Buhl, ID, USA.

^d^Omega Protein Inc., Reedville, VA, USA.

^e^De‐hulled solvent‐extracted soybean meal, Bunge Limited, Decatur, AL, USA.

^f^Corn protein concentrate, Empyreal 75TM, Cargill Corn Milling, Cargill, Inc, Blair, NE, USA.

^g^The Solae Company, St. Louis, MO, USA.

^h^Alfa Aesar, Haverhill, MA, USA.

^i^MP Biomedicals, Solon, OH, USA.

^j^Trace mineral premix (g/100 g premix): cobalt chloride, 0.004; cupric sulfate pentahydrate, 0.550; ferrous sulfate, 2.000; magnesium sulfate anhydrous, 13.862; manganese sulfate monohydrate, 0.650; potassium iodide, 0.067; sodium selenite, 0.010; zinc sulfate heptahydrate, 13.193; alpha‐cellulose, 69.664.

^k^Vitamin premix (g/kg premix): thiamin HCl, 4.95; riboflavin, 3.83; pyridoxine HCl, 4.00; Ca‐pantothenate, 10.00; nicotinic acid, 10.00; biotin, 0.50; folic acid, 4.00; cyanocobalamin, 0.05; inositol, 25.00; vitamin A acetate (500,000 IU/g), 0.32; vitamin D3 (1,000,000 IU/g), 80.00; menadione, 0.50; alpha‐cellulose, 856.81.

^l^VWR International, Radnor, PA, USA.

^m^Stay C, (L‐ascorbyl‐2‐polyphosphate, 35% active vitamin C), Roche Vitamins Inc., Parsippany, NJ, USA.

^n^Beantown Chemical, Hudson, NH, USA.

^o^AB Vista, Woodstock Court, Blenheim Road, Business Park, Marlborough, Wiltshire, SN8 4AN.

^p^Alltech, Nicholasville, Kentucky, USA.

^q^Proximate analyses were conducted by Midwest Laboratories, Omaha, NE, USA.

Dry ingredients, including enzymes, were thoroughly mixed using an ingredient mixer (Globe Food Equipment Co., Moraine, OH, USA). Afterwards, fish oil and soy oil were added into the mixture, followed by the addition of warm water (35%–45% of dry ingredients weight) into the mash to achieve a suitable consistency for pelleting. Each diet was cold‐extruded using a meat grinder equipped with a 3‐mm die to obtain the desired pellet size. Finally, the feed was oven‐dried at 34°C. Econase XT (i.e., XYL) is reported by the manufacturer to be intrinsically thermostable up to 95°C, while the enzyme activities of Allzyme SSF (i.e., MES) have been shown to remain stable during pelleting and drying at 55°C [22]. Therefore, the cold‐pelleting and low‐temperature drying conditions used in the present study were within the reported thermal stability ranges of both enzyme products. The proximate composition of all diets was analyzed from Midwest Laboratories (Omaha, NE, USA) following standard protocols [23].

### 2.2. Growth Trial

This experiment was conducted at E.W. Shell Fisheries Center, Auburn University (Auburn, AL, USA). The 5‐week growth trial was performed in a recirculating aquaculture system using clear water with no natural productivity. Salinity was maintained at approximately 12 g/L by adding manufactured sea salt (Instant Ocean, Blacksburg, VA). Water quality parameters, such as dissolved oxygen (DO), salinity, and temperature, were measured twice daily (7:00 and 15:00) using a handheld Pro 2030 (YSI Inc., Yellow Springs, OH, USA). pH was measured twice per week using a handheld pH‐meter (PH110: ExStik Refillable pH Meter, Extech Instruments., Nashia, NH, USA). Total ammonia nitrogen (TAN) and nitrite nitrogen were measured twice per week using a YSI 9500 photometer (YSI Inc., Yellow Springs, OH, USA). Water quality parameters were measured as (mean ± standard deviation, SD): temperature 28.62 ± 0.56°C, DO 7.30 ± 0.48 mg/L, salinity 12.12 ± 1.72 g/L, pH 8.03 ± 0.13, TAN 0.19 ± 0.14 mg/L, and nitrite 0.10 ± 0.10 mg/L. During the experimental trial, the water quality parameters were maintained within acceptable ranges for the Pacific white shrimp culture [22].

Shrimp juveniles were obtained from Homegrown Shrimp USA (Indiantown, FL, USA) and raised in a biofloc system until the desired size. At stocking, 10 shrimp were size‐sorted for uniformity and group‐weighed and stocked at an average initial weight of 0.72 ± 0.03 g (mean ± SD) in 108 L identical aquaria (~28 shrimp/m^2^). Each treatment with six replicate aquaria was randomly assigned to shrimp. Daily feed rations were given four times a day (7:00, 11:00, 15:00, and 17:00) and calculated based on a pre‐programmed standard feeding protocol that assumes shrimp double their weight weekly up to 1 g, then gain 0.8 g per week for the remainder of the trial with an assumed feed conversion ratio (FCR) of 1.8. Shrimp were counted weekly to estimate survival and adjust feed inputs.

### 2.3. Sampling and Calculations

At termination, all shrimp were counted and group‐weighed to determine survival, growth performance parameters, and feed utilization. Four shrimp per replicate aquarium were preserved at −20°C for crude protein analysis. At the beginning of the feeding trial, shrimp samples were also stored at −20°C for crude protein analysis. Additionally, hepatopancreas samples from three shrimp per tank were collected from four random replicate tanks per treatment and preserved in the DNA/RNA Shield (ZYMO Research, Irvine, CA, USA) at −80°C for gene expression analysis. Nitrogen content from whole‐body shrimp was determined by drying shrimp at 95°C until constant weight was obtained. Subsequently, dry samples were ground and analyzed using a nitrogen analyzer (LECO FP828P, LECO Corporation., Saint Joseph, MO, USA) for crude protein content estimations, which were calculated by multiplying nitrogen by 6.25 to estimate the protein content. The performance variables and feed calculation were estimated as follows:

Survival %= Number of shrimp at terminationNumber of shrimp stocked×100,

Final mean weight g= Total biomass gNumber of shrimp at termination,

Final biomass g=Total weight of all shrimp in each aquaria at termination,

Weight gain %= Final mean weight g−initial mean weight gInitial weight g×100,

Weekly weight gain g= Final mean weight g−initial mean weight gCulture period weeks,

Thermal-unit growth coefficient TGC= Final weight13−initial weight13Temperature °C×days ×100,

Feed conversion ratio FCR= Feed fed for the entire study gFinal biomass gained during study g×100 ,

Apparent net protein retention %= Final CP content % ×final mean weight g−initial CP content %×initial mean weight gTotal feed offered g ×CP ofthe feed %.

### 2.4. Digestibility Trial

A digestibility trial was conducted in the same RAS as previously described. The same dietary formulations from the growth trial were used, albeit they contained a 1% addition of Yttrium oxide (Y_2_O_3_) (Thermo Fisher Scientific., Ward Hill, MA, USA) which was added as an inert marker. Eight shrimp with an average individual weight of 9.69 ± 0.51 g (mean ± SD) were stocked in 83.9‐L aquaria (~29 shrimp/m^2^). Each dietary treatment consisted of three replicate aquaria. Shrimp were acclimated to their assigned 1% yttrium oxide‐supplemented diet for 3 days before collecting fecal matter. Shrimp were offered feed four times throughout the day in slight excess. 1 h post feeding, feces were siphoned and collected. The first daily fecal collection was discarded, whilst the subsequent fecal collections were rinsed with deionized water and dried in an oven at 95°C overnight. Feces were collected for 3 days, after which a sample quantity appropriate for analysis was obtained. Throughout the feces collections, water quality parameters were recorded as mentioned above and kept as follows (mean ± SD): temperature 28.58 ± 0.51°C, DO 7.32 ± 0.36 mg/L, salinity 13.07 ± 0.20 g/L, pH 7.99 ± 0.08, TAN 0.17 ± 0.07 mg/L, and nitrite 0.12 ± 0.12 mg/L.

The gross energy of both the experimental diets and the feces collected was analyzed using a semi‐micro bomb calorimeter (Model 6725, Parr Instrument Company., Moline, IL, USA). Yttrium concentrations in the experimental diets and feces were determined by the University of Missouri Agricultural Experiment Station Chemical Laboratories (Columbia, MO, USA). Following nitric acid digestion, yttrium was quantified using inductively coupled plasma‐atomic emission spectrometry (ICP‐AES) according to Anderson [24]. The apparent digestibility coefficient (ADC) of dry matter (ADDM), crude protein (ADCP), and gross energy (ADE) were calculated according to Cho et al. [25] on a dry matter basis as follows:

ADDM %=100− 100 ×%Yttrium oxide in feed%Yttrium oxide in feces,

ADCP or ADE %=100− 100×%Yttrium oxide in feed%Yttrium oxide in feces × %Nutrient in feces %Nutrient in feed .

### 2.5. Gene Expression Analysis

#### 2.5.1. Total RNA Extraction and cDNA Synthesis

Thawed samples were transferred into 96‐deep well plates containing 0.1 mm ceramic beads (Omni International, Kennesaw, GA, USA), 100 μL lysis buffer (MagMAX *mir*Vana Total RNA Isolation Kit, ThermoFisher Scientific, Waltham, MA, USA), and 0.2 μL 2‐mercaptoethanol (VWR Life Sciences USA). Samples were homogenized using a Bead Ruptor 96 (Omni International) at 30 Hz, alternating 45 s of homogenization with 45 s of rest. A total of 100 μL of homogenized hepatopancreas samples was extracted and purified using the aforementioned total RNA kit within a KingFisher Apex system (ThermoFisher Scientific Inc.). Following purification, RNA concentration and quality were measured using a NanoDrop One^C^ (Thermo Fisher Scientific Inc.). The RNA samples (50 ng/μL) were stored at −80°C until being used for cDNA synthesis.

Total RNA was converted to cDNA using the High‐Capacity cDNA Reverse Transcription Kit (Applied Biosystems, Waltham, MA, USA) following the manufacturer’s protocol. The reaction was performed using a Bio‐Rad T100 thermal cycler (Bio‐Rad Inc., Hercules, CA, USA). The cDNA samples were then diluted to reach a target concentration of 1.25 ng/μL for use in quantitative PCR (qPCR) reactions.

#### 2.5.2. qPCR

For qPCR, ribosomal protein l21 (*l21*) and β‐actin (*actb*) were selected as reference genes, while eight target genes were analyzed, as detailed in Table 2. Prior to primer efficiencies were determined via five serial dilutions (dilution factor of 2) of 10 pooled cDNA samples and calculated as:

**Table 2:** Primers used in gene expression analysis for Pacific white shrimp (*L. vannamei*) fed experimental diets containing barley as a replacement of wheat, with or without enzyme supplementation for 5‐weeks.

Genes		Sequences (5^′^–3^′^)		Efficiency (%)	Amplicon size (bp)	NCBI GenBank accession	References
Housekeeping genes							
* l21*	Ribosomal protein l21	Forward	GTTGACTTGAAGGGCAATG	98.45	246	XM_027359927	[25]
		Reverse	CTTCTTGGCTTCGATTCTG				
* actβ*	β‐actin	Forward	CATCACCAACTGGGACGACATGGA	90.76	68	XM_027367204	[26]
		Reverse	GAGCAACACGGAGTTCGTTGT				
Target genes							
* tor*	Target of rapamycin	Forward	TGCCAACGGGTGGTAGA	90.64	181	XM_027372359	[27, 28]
		Reverse	GGGTGTTTGTGGACGGA				
* ghsr1l*	Growth hormone secretagogue type 1‐like	Forward	AATTTGCGCTTGCACTACGG	97.18	100	XM_070121640	[29]
		Reverse	ATCCGGTTGAGGTGTAGCTG				
* sod*	Superoxide dismutase	Forward	TGCCACCTCTCAAGTATGATTTC	97.39	130	DQ298208	[30]
		Reverse	TCAACCAACTTCTTCGTAGCG				
* cat*	Catalase	Forward	AGAGGGTTGTGCATGCTAAG	114.45	159	XM_070119527	[31]
		Reverse	CAGCTGATCCACTCTCACCT				
* tgfβ*	Transforming growth factor β	Forward	AACCATGCCCTTGTGCAAAC	102.34	72	Y11725	[26]
		Reverse	CTTTGGGGGAACCTCGGTC				
* propo*	Prophenoloxidase	Forward	CGGTGACAAAGTTCCTCTTC	114.53	122	EF115296	[32]
		Reverse	GCAGGTCGCCGTAGTAAG				
* lgbp*	β‐1,3‐glucan binding protein	Forward	CATGTCCAACTTCGCTTTCAGA	92.00	64	XM_070119658	[33]
		Reverse	ATCACCGCGTGGCATCTT				
* hsp70*	Heat shock protein 70	Forward	CCTCCTACGTCGCCTTCACAGACA	83.91	233	AY645906	[28]
		Reverse	GGGGTAGAAGGTCTTCTTGTCTCCC

Efficiency %=10−1Slope−1× 100.

The qPCR was performed in a CFX Opus 384 Real‐Time PCR System (Bio‐Rad Inc.). Each 10 μL reaction consisted of 5 μL SsoAdvanced Universal SYBR Green Supermix (Bio‐Rad Inc.), 0.5 μL of each forward and reverse primer (10 μM stock), 4 μL of diluted cDNA sample (5 ng total), subjected to 40 cycle settings of 95°C for 120 s, 95°C for 15 s, 60°C for 30 s, and 72°C for 30 s, followed by melt curve analysis to verify the specificity of the amplified products. During the melt curve step, the reaction temperature was increased from 62 to 95°C at a continuous ramp rate of 0.05°C/s. A single, sharp peak in the derivative plot (‐dF/dT) was used to confirm the presence of a specific amplicon. All relative quantifications were first normalized against the geometric average of the reference genes. Each value was then compared to the basal treatment group (BW, relative expression = 1) using the 2^−ΔΔCt^ method [26, 27].

### 2.6. Statistical Analysis

Growth and digestibility data were analyzed using Statistical Analysis System (SAS) for Windows (V9.4, SAS Institute, Cary, NC, USA) through one‐way analysis of variance (ANOVA). Prior to analysis, assumptions of normality, and homogeneity of variances were measured using the Shapiro–Wilk test and Bartlett’s test, respectively. When a significant effect was detected (*p* < 0.05), differences among means were separated using the Student–Newman–Keuls (SNK) post hoc test. For gene expression, data were log‐transformed and analyzed through one‐way ANOVA in R (version 4.4.0) to test for significant variation at the 5 % probability level (*p* < 0.05). The assumptions of normality and homogeneity of variance were checked using the Shapiro–Wilk test on model residuals and Levene’s test, respectively. All the values are expressed as the mean ± standard error (*n* = 4). For a multigene expression pattern, the treatment means were standardized (*Z*‐score), and hierarchical clustering based on Euclidean distance and Ward’s minimum variance method was performed to visualize the similarities and divergence in overall hepatopancreatic gene expressions among different dietary treatments.

## 3. Results

### 3.1. Growth Performance

During the experimental trial, water quality parameters were maintained within acceptable ranges for the Pacific white shrimp culture (Table 3) [34]. No significant differences were found in any growth parameters measured among shrimp offered the various experimental diets (Table 4; *p* > 0.05). Survival ranged from 80.0% in the BB and BB:MES‐L treatments to 90.0% in the BB:XYL‐L treatment. In terms of final mean weight, the BB:XYL‐L treatment was numerically higher (7.79 g) and lower in the BB treatment (7.12 g). A numerically higher final biomass was recorded in the BB:XYL‐L treatment (70.05 g), while the lowest was observed in the BB treatment (56.21 g). Similarly, the highest percent weight gain was observed in the BB:XYL‐L treatment (1006.0 %), while the lowest was observed in the BB:MES‐H treatment (898.9%). Despite this range, none of the differences were statistically significant (*p* > 0.05). Thermal growth coefficient was consistent across all treatments, ranging from 0.22 in BB:MES‐L and BB:XYL‐L treatments to 0.26 in BW treatment.

**Table 3:** Summary of water quality parameters in a clear water recirculating system used in a diet trial for Pacific white shrimp over 5‐weeks.

Parameters	Values
Dissolved oxygen (mg/L)	7.30 ± 0.48 (6.35, 8.45)
Temperature (°C)	28.62 ± 0.56 (27.60, 29.80)
Salinity (mg/L)	12.12 ± 1.72 (10.00, 14.70)
Total ammonia nitrogen (mg/L)	0.19 ± 0.14 (0.00, 0.41)
Nitrite (mg/L)	0.10 ± 0.10 (0.03, 0.34)
pH	8.03 ± 0.13 (7.76, 8.16)

*Note:* Values are shown as the mean ± standard deviation.

**Table 4:** Growth performance of Pacific white shrimp (*L. vannamei*) fed experimental diets containing barley as a replacement of wheat, with or without enzyme supplementation for 5‐weeks.

Treatments^a^	Survival (%)	Final mean weight (g)	Final Biomass (g)	Weight gain (%)	Weekly weight gain (g)	TGC	FCR	ANPR (%)
BW	83.33	7.54	62.96	955.6	1.32	0.26	2.22	20.39
BB	80.00	7.12	56.21	915.6	1.24	0.25	2.37	17.20
BB:XYL‐L	90.00	7.79	70.05	1006.0	1.37	0.22	2.15	20.00
BB:XYL‐H	81.66	7.36	60.17	931.9	1.29	0.23	2.34	17.46
BB:MES‐L	80.00	7.50	59.99	912.9	1.31	0.22	2.33	18.32
BB:MES‐H	83.33	7.20	59.95	898.9	1.26	0.24	2.39	18.09
*p*‐Value^b^	0.701	0.599	0.230	0.567	0.617	0.342	0.546	0.201
PSE^c^	4.80	0.28	3.86	44.03	0.05	0.01	0.10	5.34

Abbreviations: ANPR, apparent net protein retention; FCR, feed conversion ratio; TGC, thermal‐unit growth coefficient.

^a^Treatments: BW, wheat‐based diet; BB, barley‐based diet; BB:XYL‐L, barley‐based diet supplemented with 0.01% econase XT25; BB:XYL‐H, barley‐based diet supplemented with 0.02% econase XT25; BB:MES‐L, barley‐based diet supplemented with 0.05% allzyme SFF; and BB:MES‐H, barley‐based diet supplemented with 0.1% allzyme SFF.

^b^One‐way ANOVA, significant differences among the means were analyzed using Student–Neuman–Keuls (SNK) means separation test.

^c^PSE, pooled standard error, *n* = 6.

The BB:XYL‐L treatment presented the highest weekly weight gain (1.37 g), while the BB treatment had the lowest (1.24 g). FCR values ranged from 2.15 in the BB:XYL‐L treatment to 2.37 and 2.39 in the BB and BB:MES‐H treatments, respectively. However, ANPR ranged from 17.20% in the BB to 20.39% in the BW treatment. Differences in these parameters were not statistically significant (*p* > 0.05).

### 3.2. Nutrient Digestibility

The results of nutrient digestibility analysis are presented in Table 5. ADDM in diets ranged from 35.25% to 49.09%. ADDM of BB, BB:XYL‐H, and BB:MES‐H was significantly lower than that of BW (*p* < 0.05). The ADE in the evaluated treatments ranged from 44.63% to 60.38%. Briefly, the ADE of BW and BB:XYL‐L were significantly higher than those of BB, and BB:XYL‐H diets (*p* < 0.05). The ADCP ranged from 52.08% in Barley treatment to 66.03% in the BB:MES‐L group. Enzyme supplemented treatments were numerically higher than BB un‐supplemented treatments, however, no significant differences were observed in ADCP (*p* > 0.05).

**Table 5:** Apparent digestibility coefficients of dry matter (ADDM), energy (ADE), and protein (ADCP) of the various diets offered to Pacific white Shrimp (*L. vannamei*) fed experimental diets containing barley as a replacement of wheat, with or without enzyme supplementation for 5‐weeks.

Treatments^1^	ADDM (%)	ADE (%)	ADCP (%)
BW	49.09^a^	60.38^a^	63.26
BB	36.27^b^	46.68^b^	52.08
BB:XYL‐L	43.82^ab^	59.20^a^	62.01
BB:XYL‐H	35.96^b^	44.63^b^	59.28
BB:MES‐L	44.20^ab^	56.26^ab^	66.03
BB:MES‐H	35.25^b^	51.16^ab^	56.15
*p*‐Value^2^	0.001	0.010	0.238
PSE^3^	1.98	2.84	3.77

*Note:* Values in the same column with different superscript letters are significantly different (*p* < 0.05).

^1^Treatments: BW, wheat‐based diet; BB, barley‐based diet; BB:XYL‐L, barley‐based diet supplemented with 0.01% Econase XT25; BB:XYL‐H, barley‐based diet supplemented with 0.02% Econase XT25; BB:MES‐L, barley‐based diet supplemented with 0.05% allzyme SFF; and BB:MES‐H, barley‐based diet supplemented with 0.1% allzyme SFF.

^2^One‐way ANOVA, significant differences among the means were analyzed using Student–Neuman–Keuls (SNK) means separation test.

^3^PSE, pooled standard error, *n* = 3.

### 3.3. Hepatopancreatic Gene Expression

#### 3.3.1. Growth Regulation and Redox Homeostasis

Although not significant (*p* > 0.05), the expression pattern of *tor* showed that BW and BB:MES‐L had similar expression levels along with relative upregulation in BB:MES‐H (Figure 1a). However, BB, BB:XYL‐L, and BB:XYL‐H showed relatively lower downregulation compared with that of other treatments (Figure 1a). In the case of *ghsr1l*, BB:XYL‐L and BB:XYL‐H showed the lowest downregulation (*p* > 0.05), and BB:MES‐H showed the most similarity with BW treatment (Figure 1b).

**Figure 1:**
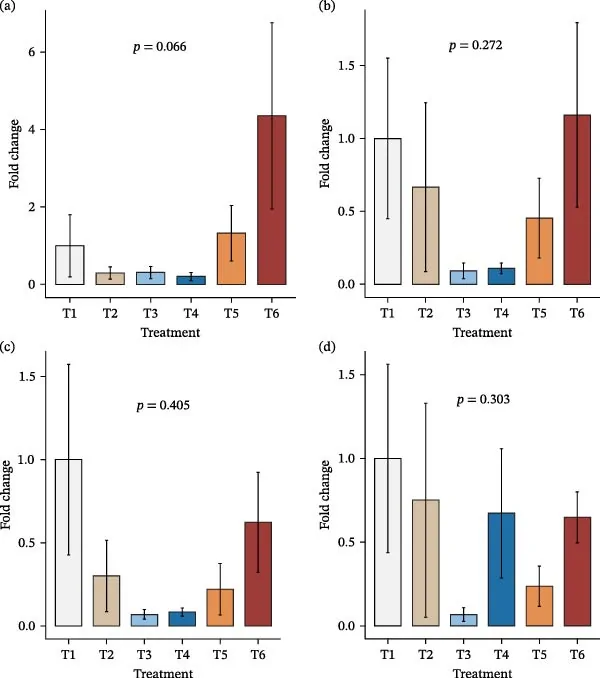
Expression of (a) target of rapamycin (*tor*), (b) growth hormone secretagogue type 1‐like (*ghsr1l*), (c) superoxide dismutase, and (d) catalase (*cat*) in the hepatopancreas of Pacific white shrimp fed different carbohydrate sources for 5‐weeks. All the values are expressed as mean ± standard error (*n* = 4). Treatments: T1, wheat‐based diet (BW); T2, barley‐based diet (BB); T3, barley‐based diet supplemented with 0.01% econase XT25 (BB:XYL‐L); T4, barley‐based diet supplemented with 0.02% econase XT25 (BB:XYL‐H); T5, barley‐based diet supplemented with 0.05% allzyme SFF (BB:MES‐L); and T6, barley‐based diet supplemented with 0.1% allzyme SFF (BB:MES‐H).

#### 3.3.2. Innate Immune Response and Cellular Stress

None of the innate immune response genes, *tgfβ*, *propo*, or *lgbp*, showed a significant (*p* > 0.05) difference among dietary treatments (Figure 2a,b,c, respectively). However, all genes showed a clear pattern of expression, with BB:MES‐H exhibiting a level of expression similar to that of BW, whereas BB:XYL‐L and BB:XYL‐H were the most downregulated treatments (Figure 2a,b,c). The cellular stress gene *hsp70* showed no significant difference (*p*  > 0.05) and exhibited relatively high expression in BB:XYL‐H (Figure 2d).

**Figure 2:**
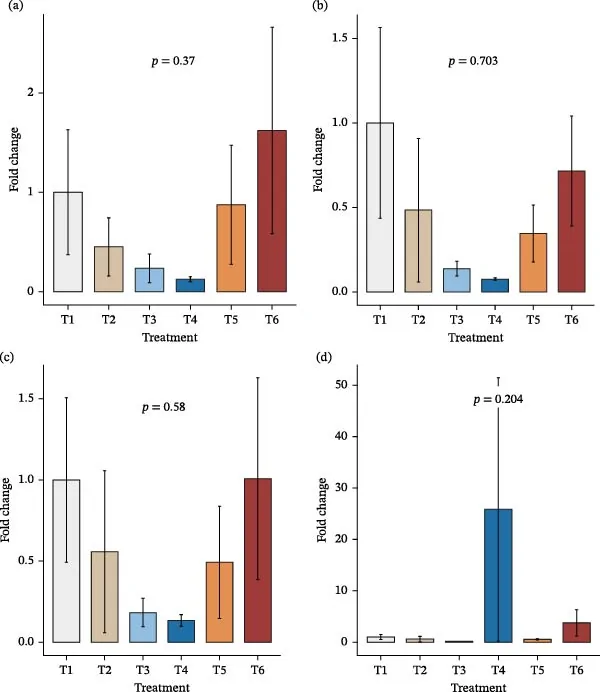
Expression of (a) transforming growth factor β (*tgfβ*), (b) prophenoloxidase (*propo*), (c) lipopolysaccharide and β‐1,3‐glucan binding protein (*lgbp*), and (d) heat shock protein 70 (*hsp70*) in the hepatopancreas of Pacific white shrimp fed different carbohydrate sources for 5‐weeks. All the values are expressed as mean ± standard error (*n* = 4). Treatments: T1, wheat‐based diet (BW); T2, barley‐based diet (BB); T3, barley‐based diet supplemented with 0.01% econase XT25 (BB:XYL‐L); T4, barley‐based diet supplemented with 0.02% econase XT25 (BB:XYL‐H); T5, barley‐based diet supplemented with 0.05% allzyme SFF (BB:MES‐L); and T6, barley‐based diet supplemented with 0.1% allzyme SFF (BB:MES‐H).

#### 3.3.3. Integrated Multigene Response

Hierarchical clustering of standardized gene expression profiles indicated distinct physiological response groups among dietary treatments (Figure 3a). BW, BB:MES‐L, and BB:MES‐H showed the most similar expression profile of different hepatopancreatic genes, whereas the other three treatments, BB, BB:XYL‐L, and BB:XYL‐H, made different groups showing relatively lower expression levels (Figure 3a). Moreover, the Euclidean distance matrix showed that BB:XYL‐L was the most different treatment in terms of the overall gene expression pattern from the basal treatment, BW, followed by BB:XYL‐H (Figure 3b). The cladogram in both figures (Figure 3a,b) showed that BW and BB:MES‐L had the most similar expression pattern among themselves than the other treatments.

**Figure 3:**
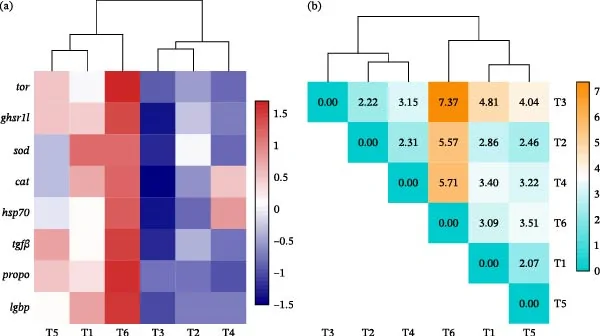
(a) Multigene hepatopancreatic transcriptional profiles (*Z*‐score) and hierarchical clustering across dietary treatments, and (b) Euclidean distance matrix with hierarchical clustering illustrating multigene transcriptional divergence among diets fed to Pacific white shrimp for 5‐weeks. Treatments: T1, wheat‐based diet (BW); T2, barley‐based diet (BB); T3, barley‐based diet supplemented with 0.01% econase XT25 (BB:XYL‐L); T4, barley‐based diet supplemented with 0.02% econase XT25 (BB:XYL‐H); T5, barley‐based diet supplemented with 0.05% allzyme SFF (BB:MES‐L); and T6, barley‐based diet supplemented with 0.1% allzyme SFF (BB:MES‐H).

## 4. Discussion

Carbohydrates serve as an economical energy source in shrimp diets, resulting in protein‐sparing effects, and their inclusion is desirable due to their binding properties [3, 4]. Efficiency of carbohydrates utilization is highly species‐ and formulation‐dependent. Several studies have demonstrated that wheat could support optimal growth in different species such as Florida pompano [35], Nile tilapia (*Oreochromis niloticus*) [36], and Pacific white shrimp [2]. Nevertheless, decreasing the reliance on common cereal grains for other advantageous ingredients, such as barley, that could partially or fully replace wheat in shrimp diets without compromising growth is crucial. Nonetheless, high levels of ANF, such as β‐glucans and arabinoxylans, are a potential disadvantage in barley; therefore, carbohydrase supplementation is commonly used to overcome increased NSP levels. In the current study, two different types of exogenous enzymes were used, that is, XYL and MES at low and high doses in barley‐based diets. Although barley contains more β‐glucan than wheat, a standalone β‐glucanase treatment was not included in the present study. However, MES provided β‐glucanase activity (200,000 IU/kg) together with other exogenous enzymes as part of the multienzyme mixture [21].

In the present study, the replacement of wheat with barley and the supplementation of carbohydrases were evaluated in shrimp diets. In terms of growth, no statistically significant differences (*p* > 0.05) were observed among dietary treatments for any of the measured parameters under the reported conditions. Minor adjustments in corn starch across diets to maintain isonitrogenous and isolipidic conditions are unlikely to have influenced the primary comparisons as all barley‐based diets contained similar levels. Results indicate that barley can replace wheat in shrimp diets without compromising the growth. Interestingly, BB:XYL‐L showed numerically higher growth response than other groups, which may be due to the presence of XYL in the diet which helped them utilize the diet more efficiently as evident from the digestibility. Similar to our findings, dietary inclusion of barley showed no impairment in terms of growth in pigs and broilers [37, 38]. Pinedo‐Gil et al. [39] reported that dietary inclusion of barley at up 32% sustained optimal growth parameters in rainbow trout. While Shahzad et al. [40] indicated that replacing up to 20% of fishmeal with dietary barley seed meal is viable without adversely affecting growth parameters in common carp (*Cyprinus carpio*). The inclusion of barley flour containing different levels of β‐glucan (38, 52, and 82 g/kg) as a replacement for wheat flour did not substantially affect weight gain in this species [41]. Differences in study outcomes may be influenced by several factors including animal species, carbohydrases and their doses, inherent physiological variation in digestive environments, origin and composition of the plant‐based ingredients, and pH. Enzymes, such as XYL, have an optimal pH range, and deviations from this range can alter the enzyme structure and reduce catalytic activity [42].

ADDM represents the total level of nutrients digested since feed components are not digested by the animal in equal proportions [43]. ADDM in the BW diet was significantly higher than that in BB, BB:XYL‐H, and BB:MES‐H, which indicated that the nutrients from the BW treatment could be better digested and absorbed by shrimp. Similar results were found in olive flounder (*Paralichthys olivaceus*) fed wheat flour [44]. Compared to wheat, barley is often associated with a higher β‐glucan content and for its higher levels of NSP, which are complex to digest. Toghyani et al. [45] also reported that complex carbohydrates have a lower ADDM than simple carbohydrates.

Additionally, an improvement in ADE in BB:XYL‐L compared with the BB diet was observed (*p* < 0.05). These results could be potentially attributed to XYL, which hydrolyzes NSP such as arabinoxylans, reducing digesta viscosity, releasing entrapped energy in cell walls, and facilitating the absorption of other nutrients [17, 46]. Our findings are consistent with previous research in other species, such as swine and poultry fed barley‐based diets supplemented with β‐glucanase or XYL, which showed that carbohydrase inhibit antinutritional effects of both β‐glucans and arabinoxylans in barley‐based diets [45, 47]. Similar results have been observed in Nile tilapia fed a sunflower meal, rapeseed meal, and wheat distillers dried grains with solubles (DDGS)‐based diet with high NSP content, supplemented with XYL (Danisco XYL, Danisco Animal Nutrition, Marlborough, UK) at 4000 U/kg [48]. Similarly, Hassaan et al. [18] reported that the inclusion of XYL in fish meal and sunflower meal‐based diets improved energy digestibility in the same species. In rainbow trout, gross energy digestibility was enhanced by supplementing hemicelluloses to a lupin‐based diet [49]. In contrast, ADE were not statistically different in Florida pompano, *Trachinotus carolinus*, fed a plant‐based diet supplemented with a carbohydrase enzyme complex (Natugrain TS L, BASF Corporation, Ludwigshafen, Germany) which contained endo‐1,4‐ß‐XYL and endo‐1,4‐ß‐glucanase [20]. Similarly, Qiu and Davis [17] reported that the inclusion of carbohydrase (Rovabio Excel LC, Adisseo., Alpharetta, GA, USA), whose main enzymatic activity is XYL and β‐glucanase, showed no effect on energy digestibility in Pacific white shrimp at 0.01%, 0.02%, 0.03%, 0.04%, and 0.05% of inclusion.

Enzyme supplementation enhanced numerically ADCP in all treatments. BB:MES‐L presented a numerically higher value (66.03%) compared to BW (63.26%); however, no statistically significant differences were observed in ADCP, which aligns with the lack of significance in ANPR. In contrast, carbohydrases supplementation in diets of different species, such as Nile tilapia [18], Florida pompano [20], and Pacific white shrimp [17], showed improvements in apparent protein digestibility. A possible explanation for those results is that exogenous enzymes improve ADCP by increasing the contact between the digesta and proteases; therefore, digestive protease gain access to proteins [50]. A limitation of the present study was the absence of a treatment supplemented specifically with β‐glucanase. Although MES contained substantial β‐glucanase activity along with other enzymes, its effects could not be attributed specifically to β‐glucanase because of the combined activities of the multienzyme mixture. Therefore, the current study could not determine whether targeted hydrolysis of barley β‐glucans would further improve nutrient and energy digestibility in shrimp. Future studies should evaluate β‐glucanase alone and at graded supplementation levels to determine its specific efficacy in barley‐based shrimp diets.

Evaluating gene expression of growth‐regulation‐ and redox homeostasis‐related genes, including *tor*, *ghsr1l*, *sod*, and *cat*, provides a mechanistic understanding of shrimp’s physiological responses. The mechanistic target of rapamycin (mTOR) signaling pathway is recognized as a complex growth‐regulating network that regulates metabolic activity, protein synthesis, and cellular growth in response to nutrient availability [51]. Similarly, growth hormone‐related genes have been targeted for their key physiological roles in growth regulation, development, and metabolism [52]. On the other hand, to prevent damage from excessive reactive oxygen species (ROS) and reactive nitrogen species (RNS) that are released by the metabolism of carbohydrates, protective antioxidant enzymatic and nonenzymatic systems, including SOD and CAT, are activated [53, 54]. In this trial, the absence of significant differences in the hepatopancreatic mRNA levels of growth‐related genes (*propo* and *gh*) suggests that barley and/or enzyme supplementation did not disrupt the normal growth‐signaling status of shrimp. In contrast to previous studies, transcriptional changes in insulin‐like growth factor (IGI‐I; a growth‐related hormone) were significantly affected in plant‐based diets containing de‐oiled rice bran (DORB) in rohu (*Labeo rohita*) diets [55]. Additionally, we found no statistically significant differences in oxidative response‐related genes (*sod* and *cat*), which suggests that barley inclusion did not induce oxidative stress in shrimp and is therefore safe as a feed ingredient. Conversely, *sod* and *cat* expression was significantly affected by the carbohydrate source in largemouth bass (*Micropterus salmoides*) diets [56]. Prophenoloxidase (ProPO) is converted into PO through the ProPO‐activating enzyme (ppA), which, along with hemocytes, plays a vital role in melanin production via the ProPO system [57, 58]. The ProPO system can be activated by *hsp70*, which acts as a damage‐associated molecular pattern (DAMP) by interacting with *lgbp*, aiding in melanization [59]. Both melanin and hemocytes contribute significantly to immune defense in crustaceans [60]. The *hsp70* also enhances anti‐inflammatory cytokine production, responds to thermal stress, and has been associated with immune stimulation in crustaceans. At the same time, *lgbp* plays an important role in innate immune recognition [59]. On the other hand, transforming growth factor beta (*tgfβ*) encodes anti‐inflammatory cytokines, promotes homeostasis, and regulates immune responses [61]. In the present study, hepatopancreatic gene expression showed no significant differences across the observed immune‐related genes. These results suggest that neither immunological augmentation nor impairment occurred in unsupplemented and supplemented barley diets. Barley is well known for its high levels of β‐glucans. The hierarchical clustering supported that barley‐based diets supplemented with allzyme SFF containing β‐glucanase showed the most similar overall gene expression to the wheat‐based basal diet. Although studies evaluating the immune response of shrimp fed barley diets are scarce, several have focused on the role of β‐glucans in modulating immune responses. No significant differences were found in salmon (*Salmo salar*) fed β‐glucan‐containing diets in gene expression of immune‐related genes, including *tgfβ* [62]. On the other hand, the *propo* expression remained unaffected in Pacific white shrimp fed plant‐based diets [63]. Conversely, the expression levels of *lgbp* were significantly increased in juvenile prawns (*Macrobrachium nipponense*) fed β‐1,3‐glucan‐supplemented diets [64]. Similar results were found in Pacific white shrimp, in which the intestinal expression of *lgbp* and *hsp70* was enhanced when fed β‐1,3‐glucan, suggesting an improvement in immunity parameters [65]. Overall, gene expression did not differ significantly among treatments. The multigene analysis showed only numerical similarities, with Allzyme SSF containing β‐glucanase appearing closer to the wheat‐based diet; however, these trends should be interpreted cautiously, as smaller treatment effects may not have been detected.

## 5. Conclusion

In conclusion, the present work suggests that barley did not impair growth relative to wheat under the tested condition and could be used as a substitute for wheat in Pacific white shrimp diets without adverse effects on growth performance, nutrient utilization, or physiological gene expression. Additionally, low‐dose XYL supplementation improved energy digestibility, and the multienzyme supplementation yielded gene expression patterns similar to wheat‐based diets. The lack of significant differences in growth parameters may reflect high within‐treatment variability; future studies with increased replication or a longer trial duration may clarify these trends. Moreover, future studies should also quantify enzyme recovery and activity in finished diets after feed preparation and storage to further confirm the relationship between the intended supplementation levels and the observed dietary responses. Further research is needed to evaluate the efficacy of other enzymes in plant‐based diets, including barley, a major carbohydrate ingredient in Pacific white shrimp diets.

## Funding

This work was supported by the Non‐Assistance Cooperative Agreement between Auburn University and the USDA‐Agricultural Research Service (Grant 6010‐32000‐028‐001‐S) “Developing sustainable aquaponic production systems.” These studies were also partially supported by USDA National Institute of Food and Agriculture (NIFA) Hatch projects to D. Allen Davis (PI) under Grant ALA016‐1‐19102 and Timothy J. Bruce (PI) under Grant ALA016‐1‐19143.

## Disclosure

The mention of trademarks or proprietary products is for informational purposes only and does not imply endorsement by Auburn University, nor does it exclude other products or services that may be suitable.

## Ethics Statement

Animal care protocols were in compliance with the Auburn University animal care and use policies.

## Conflicts of Interest

The authors declare no conflicts of interest.

## Data Availability

The data that support the findings of this study are available from the corresponding author upon reasonable request.
